# Supplementary material for: Factors Affecting the Stability of Platinum(II) Complexes with 1,2,4-Triazolo[1,5-a]pyrimidine Derivatives and Tetrahydrothiophene-1-Oxide or Diphenyl Sulfoxide
Source: Int J Mol Sci. 2022 Mar 26;23(7):3656. doi: 10.3390/ijms23073656 (PMC8998453; doi:10.3390/ijms23073656)
Supplement: Supplementary file 1 [file ijms-23-03656-s001.zip › ijms-1581120-supplementary.pdf]

Table. Calculate Pt-NMR v Angle

	DMTP						IB[illegible]	
	cis-			**(B1)** *trans-*			*cis-*	
Torsion	Energy	Pt NMR	Torsion	Energy	Pt NMR	Torsion	Energy	Pt NMR
degree	*kcal/mol*	*ppm*	*degree*	*kcal/mol*	*ppm*	*degree*	*kcal/mol*	*ppm*
175,60	6,683	-3189,53	-179,96	0,658	-3474,61	-174,06	6,102	-3246,14
165,60	6,959	-3141,88	-169,96	0,498	-3457,71	-164,06	5,444	-3299,79
155,60	7,077	-3125,09	-159,96	0,480	-3449,8	-154,06	4,624	-3295,14
145,60	4,330	-3282,81	-149,96	0,485	-3413,21	-144,06	3,900	-3327,35
135,60	3,478	-3313,33	-139,96	0,922	-3429,86	-134,06	2,987	-3332,49
125,60	2,824	-3343,78	-129,96	1,115	-3352,93	-124,06	2,385	-3342,55
115,60	2,294	-3359,88	-119,96	1,048	-3311,64	-114,06	2,015	-3370,28
105,60	2,128	-3372,08	-109,96	0,865	-3280,01	-104,06	1,848	-3362,19
95,60	2,397	-3352,28	-99,96	1,269	-3240,44	-94,06	1,895	-3353,19
85,60	2,695	-3345,59	-89,96	1,554	-3331,85	-84,06	1,911	-3348,92
75,60	2,724	-3331,1	-79,96	1,438	-3381,45	-74,06	1,703	-3344,48
65,60	2,415	-3308,26	-69,96	1,152	-3373,53	-64,06	1,367	-3339,15
55,60	1,954	-3324,92	-59,96	0,997	-3327,53	-54,06	1,209	-3336,96
45,60	1,636	-3307,84	-49,96	0,896	-3410,28	-44,06	1,056	-3327,45
35,60	1,039	-3282,1	-39,96	0,542	-3412,43	-34,06	0,854	-3327,11
25,60	0,318	-3210,45	-29,96	0,183	-3439,45	-24,06	0,492	-3280,47
15,60	0,000	-3184,81	-19,96	0,034	-3451,67	-14,06	0,084	-3268,12
5,60	0,201	-3173,03	-9,96	0,000	-3455,77	-4,06	0,180	-3247,44
-4,40	0,095	-3261,16	0,04	0,133	-3494,24	5,94	0,040	-3170,18
-14,40	0,161	-3294,68	10,04	0,065	-3478,87	15,94	0,000	-3177,56
-24,40	0,422	-3304,4	20,04	0,048	-3457,77	25,94	0,266	-3201,38
-34,40	0,922	-3335,89	30,04	0,182	-3441,81	35,94	0,996	-3286,3
-44,40	1,080	-3332,64	40,04	0,561	-3410,81	45,94	1,525	-3296,14
-54,40	1,372	-3328,16	50,04	0,931	-3401,75	55,94	1,929	-3306,64
-64,40	1,641	-3354,85	60,04	1,057	-3357,26	65,94	2,512	-3332,91
-74,40	1,796	-3356,81	70,04	0,951	-3310,85	75,94	2,665	-3334,69
-84,40	1,959	-3349,19	80,04	1,290	-3254,59	85,94	2,664	-3345,27
-94,40	1,863	-3351,13	90,04	1,628	-3301,26	95,94	2,365	-3335,41
-104,40	1,873	-3371,41	100,04	1,678	-3368,2	105,94	2,118	-3346,36
-114,40	1,983	-3369,4	110,04	0,871	-3275,73	115,94	2,244	-3331,93
-124,40	2,395	-3352,57	120,04	0,989	-3308,72	125,94	2,906	-3326,43
-134,40	2,995	-3339,07	130,04	1,197	-3313,24	135,94	3,470	-3310,38
-144,40	3,722	-3317,78	140,04	0,867	-3407,03	145,94	4,338	-3295,77
-154,40	4,603	-3307,12	150,04	0,505	-3416,45	155,94	7,067	-3107,06
-164,40	5,461	-3285,9	160,04	0,484	-3453,73	165,94	6,908	-3141,88
-174,40	6,209	-3248,28	170,04	0,505	-3461,36	175,94	6,554	-3190,11

MTP			DBTP					
**(B2)** *trans-*			*cis-*			**(B3)** *trans-*		
Torsion *degree*	Energy *kcal/mol*	Pt NMR *ppm*	Torsion *degree*	Energy *kcal/mol*	Pt NMR *ppm*	Torsion *degree*	Energy *kcal/mol*	Pt NMR *ppm*
-171,87	0,668	-3446,33	-173,76	6,540	-3239,32	-175,29	0,538	-3430,6
-161,87	0,735	-3447,93	-163,76	5,927	-3277,07	-165,29	0,423	-3424,42
-151,87	0,632	-3436,92	-153,76	5,095	-3296,37	-155,29	0,416	-3407,73
-141,87	0,745	-3399,26	-143,76	4,292	-3309,69	-145,29	0,638	-3401,7
-131,87	0,976	-3375,55	-133,76	3,328	-3342,92	-135,29	1,085	-3400,46
-121,87	1,170	-3366,63	-123,76	2,517	-3340,25	-125,29	1,275	-3348,3
-111,87	0,828	-3265,97	-113,76	2,055	-3360,22	-115,29	1,040	-3292,75
-101,87	1,105	-3237,23	-103,76	2,027	-3360,36	-105,29	1,230	-3248,3
-91,87	1,518	-3327,42	-93,76	2,093	-3366,79	-95,29	1,688	-3292,92
-81,87	1,489	-3377,54	-83,76	2,227	-3370,29	-85,29	1,715	-3364,96
-71,87	1,249	-3355,23	-73,76	2,066	-3360,89	-75,29	1,486	-3346,3
-61,87	1,010	-3318,26	-63,76	1,782	-3354,17	-65,29	1,162	-3329,68
-51,87	1,115	-3389,84	-53,76	1,576	-3342,34	-55,29	1,229	-3330,95
-41,87	0,678	-3410,72	-43,76	1,233	-3315,09	-45,29	0,844	-3408,49
-31,87	0,290	-3426,17	-33,76	0,867	-3304,48	-35,29	0,462	-3435,39
-21,87	0,056	-3434,84	-23,76	0,601	-3283,7	-25,29	0,176	-3417,99
-11,87	0,000	-3469,1	-13,76	0,271	-3273,5	-15,29	0,000	-3445,06
-1,87	0,079	-3473,23	-3,76	0,325	-3251,05	-5,29	0,055	-3437,09
8,13	0,084	-3470	6,24	0,228	-3190,72	4,71	0,067	-3438,79
18,14	0,017	-3448,04	16,24	0,000	-3171,16	14,71	0,018	-3451,1
28,14	0,122	-3427,44	26,24	0,566	-3250,93	24,71	0,110	-3424,54
38,14	0,615	-3432,42	36,24	1,013	-3287,43	34,71	0,433	-3408,79
48,14	1,066	-3407,23	46,24	1,543	-3299,86	44,71	0,833	-3408,87
58,14	1,129	-3380,69	56,24	1,944	-3307,69	54,71	1,181	-3375,63
68,14	1,006	-3290,39	66,24	2,337	-3316,12	64,71	1,035	-3287,53
78,14	1,226	-3248,27	76,24	2,622	-3322,1	74,71	1,197	-3254,16
88,14	1,584	-3294,64	86,24	2,497	-3327,12	84,71	1,623	-3265,09
98,14	1,648	-3342,97	96,24	2,311	-3348,67	94,71	1,874	-3359,83
108,14	1,341	-3350,9	106,24	2,129	-3362,9	104,71	1,691	-3368,42
118,14	0,982	-3275,97	116,24	2,344	-3335,34	114,71	1,167	-3285,62
128,14	1,189	-3328,85	126,24	3,151	-3336,24	124,71	1,216	-3311,74
138,14	1,037	-3387,27	136,24	3,847	-3331,75	134,71	1,120	-3399,94
148,14	0,679	-3415,23	146,24	7,039	-3157,91	144,71	0,809	-3410,37
158,14	0,500	-3447,25	156,24	7,405	-3148,68	154,71	0,507	-3423,89
168,14	0,649	-3468,77	166,24	7,298	-3144,51	164,71	0,464	-3447,81
178,14	0,689	-3465	176,24	7,050	-3184,88	174,71	0,604	-3447,93

DPTP					
	cis-			**(B4)** *trans-*	
Torsion	Energy	Pt NMR	Torsion	Energy	Pt NMR
degree	*kcal/mol*	*ppm*	*degree*	*kcal/mol*	*ppm*
-179,28	6,517	-3191,44	-178,38	0,549	-3454,39
-169,28	7,039	-3123,29	-168,38	0,436	-3435,81
-159,28	4,648	-3276,36	-158,38	0,332	-3431,73
-149,28	3,893	-3293,42	-148,38	0,543	-3420,76
-139,28	2,961	-3325,15	-138,38	0,889	-3403,73
-129,28	2,236	-3336,11	-128,38	1,284	-3394,75
-119,28	1,720	-3344,66	-118,38	1,054	-3302,27
-109,28	1,470	-3366,38	-108,38	1,030	-3264,08
-99,28	1,477	-3358,54	-98,38	1,390	-3265,14
-89,28	1,662	-3359,47	-88,38	1,630	-3354,4
-79,28	1,740	-3353,72	-78,38	1,470	-3387,36
-69,28	1,507	-3344,76	-68,38	1,094	-3343,33
-59,28	1,076	-3333,32	-58,38	1,063	-3338,8
-49,28	0,908	-3336,6	-48,38	0,889	-3378,2
-39,28	0,752	-3310,07	-38,38	0,538	-3422,59
-29,28	0,490	-3305,98	-28,38	0,194	-3436,9
-19,28	0,289	-3292	-18,38	0,033	-3442,03
-9,28	0,289	-3277,89	-8,38	0,068	-3448,89
0,72	0,276	-3220,83	1,62	0,060	-3447,75
10,72	0,058	-3159,61	11,62	0,000	-3442,56
20,72	0,000	-3184,48	21,62	0,019	-3425,65
30,72	0,478	-3215,23	31,62	0,313	-3448,93
40,72	1,108	-3286,78	41,62	0,789	-3438,58
50,72	1,563	-3293,48	51,62	1,251	-3433,1
60,72	2,010	-3300,71	61,62	1,177	-3303,9
70,72	2,396	-3297,59	71,62	1,086	-3270,73
80,72	2,511	-3313,69	81,62	1,439	-3249,06
90,72	2,276	-3331,92	91,62	1,800	-3332,73
100,72	1,925	-3342,42	101,62	1,824	-3364,75
110,72	1,761	-3340,64	111,62	1,551	-3356,9
120,72	2,025	-3329,99	121,62	1,434	-3390,93
130,72	2,728	-3321,67	131,62	1,408	-3412,58
140,72	3,599	-3304,72	141,62	0,999	-3435,93
150,72	4,435	-3299,97	151,62	0,650	-3456,33
160,72	5,159	-3299,97	161,62	0,513	-3463,9
170,72	5,856	-3251,09	171,62	0,535	-3468,71
